# Double Uterus (Uterus Bilocularis)

**Published:** 1882-01

**Authors:** O. Stroinski


### ARTICLE IV.

### DOUBLE UTERUS (UTERUS BILOCULARIS), ATRESIA OF THE LEFT SIDE AND GONORRHŒAL AFFECTION OF THE RIGHT.

By O. STROINSKI, M.D.

Mrs. Bertha K., a German woman, thirty-two years of age, had been married two years to a healthy man, who acquired a gonorrhœal affection one year after the marriage. She was repeatedly infected, and after three months symptoms of a severe chronic parametritis appeared. She told me that she had menstruated the first time when fifteen years of age, and without any pains or other trouble. But at the time of the next menses she began to suffer from severe pains in the suprapubic and iliac regions, and there was no flow of blood. The pains increased to a considerable degree, a high fever set in, and the physician who was treating her at this time considered her state as very dangerous, calling the disease a peritonitis. Notwithstanding several repetitions of the same symptoms, the girl recovered, and began to menstruate regularly three months after her recovery; in all, six months after the first menstruation. The menses kept on regularly for the next five years, when the same symptoms reappeared. At this time she was examined by a physician, who called the disease a "retarded menstruation," and also thought her condition very dangerous. She refused an operation then proposed.

The next eight years passed without any further accident, the menses being very regular; but after this time the sickness reappeared twice in one year—the symptoms not being so severe as formerly.

After being infected for three months (*i. e.*, nine months before I saw her) a relapse of the same disease took place, and continued for two months. In examining her, she appeared a strong and well nourished, but pale and anæmic looking woman. The external genital organs were found to be in a normal condition, and also the vagina—the latter being only somewhat shorter than usual. In entering the vagina with the index finger, a normal cervix, with a virginal *os externum*, could be felt, drawn a little to the right side; but in pushing the finger upward to the fornix vaginæ, there was found, on the left side of the cervix, a rounded body resembling exactly another but smaller cervix, but without an opening (os). Between the normal cervix and this appendix there could be distinguished a line of demarcation which continued through the whole uterus up to the fundus. In bimanual palpation the same line could be felt through the abdominal walls, and on the upper part of the fundus there was an inlet into the same, dividing the uterus in two parts. The right side was normally constructed, respecting the metritical and parametritical condition of the same, representing a normal sized pear-shaped body; but the left side was twice the size of a normal uterus, and of a rounded globular-like shape, springing like the bulk of a new growth from the former. Through the vagina and the rectum the finger found the elongation of the normal cervix as a body like that of a normal uterus, but on the left side there was a thickening of the walls and an enlargement of the uterus, beginning right behind the cervix.

By manual pressure, the walls of this side of the uterus could be compressed like a bag filled with water, but the woman fainted by this manipulation. The speculum showed the endometrium in a state of inflammation, and the os discharging a greenish-white fluid (gonorrhœal). The parametrium was very painful by touching it, and I did not introduce the sound on account of this condition. There was in this case, doubtless, a complication of three different diseases which I had to consider: *i. e.*, first, a recent gonorrhœal infection; second, a chronic parametritis; third, an occluded uterus filled with a fluid. After three months' treatment the gonorrhœa was banished entirely and the parametritis—the latter being treated by energetic painting of the posterior vaginal wall. The parts were in such a condition that I thought it advisable to open the occluded uterus. I introduced a trocar of the smallest size through the occluding membrane—which, by the way, was half an inch thick, and a dark-colored fluid, of a smell like thickened ox-gall, began to dribble away from the new os externum. I did not enlarge the small opening, but kept it in the same condition for two weeks, introducing a small bougie once or twice a day, and preventing an infection of the vagina by washing out the same three times a day with a solution of chloride of zinc. The reason for being so slow in emptying the uterus was, first, the enlarged condition of the Fallopian tube, palpable through the abdominal walls; second, the irritated state of the peritoneum, which had been inflamed so often, and which was always sensitive to the touch. In six weeks' time the uterus contracted to about the normal size, and I washed out the cavity with a strong solution of carbolic acid, the opening of the Fallopian tubes being also contracted. The sound now entered both sides of the uterus, a thick layer of (fibrous) tissue dividing the latter, and each side having the same function as the other, *i. e.*, separating the menstrual flow alternately.

The question, how was the menstrual blood in the uterus absorbed in the former menstruations?—for this must have been the case—seems to me pointed out by the irritative condition of the peritoneum covering the uterus, and I am convinced that the blood accumulated in the uterus was transuded through the walls and absorbed by the peritoneum, the process being very slow, and having been performed at several irregular intervals.

